# CD157 enhances malignant pleural mesothelioma aggressiveness and predicts poor clinical outcome

**DOI:** 10.18632/oncotarget.2186

**Published:** 2014-07-08

**Authors:** Erika Ortolan, Alice Giacomino, Francesca Martinetto, Simona Morone, Nicola Lo Buono, Enza Ferrero, Giorgio Scagliotti, Silvia Novello, Sara Orecchia, Enrico Ruffini, Ida Rapa, Luisella Righi, Marco Volante, Ada Funaro

**Affiliations:** ^1^ Laboratory of Immunogenetics, Department of Medical Sciences, University of Torino, Torino, Italy; ^2^ Department of Oncology, University of Torino, San Luigi Hospital, Orbassano, Italy; ^3^ Pathology Unit, Azienda Ospedaliera Nazionale SS. Antonio e Biagio e Cesare Arrigo, Alessandria, Italy; ^4^ Department of Surgery, Section of Thoracic Surgery, University of Torino, Torino, Italy; ^5^ Interdepartmental Center “G. Scansetti” for Studies on Asbestos and other Toxic Particulates, University of Torino, Torino, Italy

**Keywords:** mesothelioma, CD157/BST1, mTOR, tumor progression, chemotherapy resistance, prognostic marker

## Abstract

Malignant mesothelioma is a deadly tumor whose diagnosis and treatment remain very challenging. There is an urgent need to advance our understanding of mesothelioma biology and to identify new molecular markers for improving management of patients. CD157 is a membrane glycoprotein linked to ovarian cancer progression and mesenchymal differentiation. The common embryonic origin of ovarian epithelial cells and mesothelial cells and the evident similarities between ovarian and mesothelial cancer prompted us to investigate the biological role and clinical significance of CD157 in malignant pleural mesothelioma (MPM).

CD157 mRNA and protein were detected in four of nine MPM cell lines of diverse histotype and in 85.2% of MPM surgical tissue samples (32/37 epithelioid; 37/44 biphasic). CD157 expression correlated with clinical aggressiveness in biphasic MPM. Indeed, high CD157 was a negative prognostic factor and an independent predictor of poor survival for patients with biphasic MPM by multivariate survival analysis (HR = 2.433, 95% CI 1.120-5.284; *p* = 0.025). In mesothelioma cell lines, CD157 gain (in CD157-negative cells) or knockdown (in CD157-positive cells) affected cell growth, migration, invasion and tumorigenicity, most notably in biphasic MPM cell lines. In these cells, CD157 expression was associated with increased activation of the mTOR signaling pathway, resulting in decreased platinum sensitivity. Moreover, a trend towards reduced survival was observed in patients with biphasic MPM receiving postoperative platinum-based chemotherapy. These findings indicate that CD157 is implicated in multiple aspects of MPM progression and suggest that CD157 expression could be used to stratify patients into different prognostic groups or to select patients that might benefit from particular chemotherapeutic approach.

## INTRODUCTION

Malignant mesothelioma is a rare but highly aggressive tumor arising from the mesothelial cells lining the pleural, peritoneal and pericardial cavities. Exposure to asbestos is the major risk factor [1] although a latency period often exceeding four decades may pass from first exposure to disease onset [2]. Consequently, the incidence of mesothelioma is rising and will continue to rise in the coming decades as a result of past widespread exposure to asbestos [3]. Malignant pleural mesothelioma (MPM) is by far the most common form of this disease, and a vast majority of patients are diagnosed in advanced stages. By histopathology, MPM mainly consists of three distinct subtypes: epithelioid, sarcomatoid and biphasic. The epithelioid subtype is the most common, accounting for 50-60% of MPM: it is characterized by a papillary or pseudo-glandular growth pattern and has the best prognosis. The sarcomatoid subtype is the rarest form: it is characterized by a diffuse sarcomatous morphology and has the worst prognosis. The biphasic subtype accounts for 20-35% of MPM: these tumors contain both epithelial and mesenchymal components that merge into one another, likely representing an intermediate differentiation step in the epithelial-to-mesenchymal transition (EMT) process [4, 5].

Treatment of MPM is hampered by the lack of effective therapeutic options, and prognosis remains poor with a median survival of approximately 12 months [6, 7]. There is an urgent need to better understand MPM biology, to test new markers for diagnosis and prognosis, and to identify tumor targets for novel therapeutic strategies.

In the last decade, we established that CD157 (BST1), a glycosylphosphatidylinositol (GPI)-anchored member of the NADase/ADP-ribosyl cyclase gene family [8], regulates cytoskeleton reorganization and diapedesis in human leukocytes by a non-enzymatic receptor activity [9]. We also demonstrated that CD157 is expressed in epithelial ovarian cancer where it contributes to tumor progression by promoting mesenchymal differentiation [10] and is an independent prognostic factor of reduced survival [11]. Ovarian surface epithelial cells and mesothelial cells share embryonic origin, and have common biological and morphological properties. Moreover, their malignant counterparts show similar mechanisms of dissemination. The evident parallels between ovarian and mesothelial cancer prompted speculation that CD157 might also be involved in MPM. This study was undertaken to investigate the clinical relevance and biological role of CD157 in MPM.

## RESULTS

### CD157 expression and distribution in MPM

**Figure 1:**
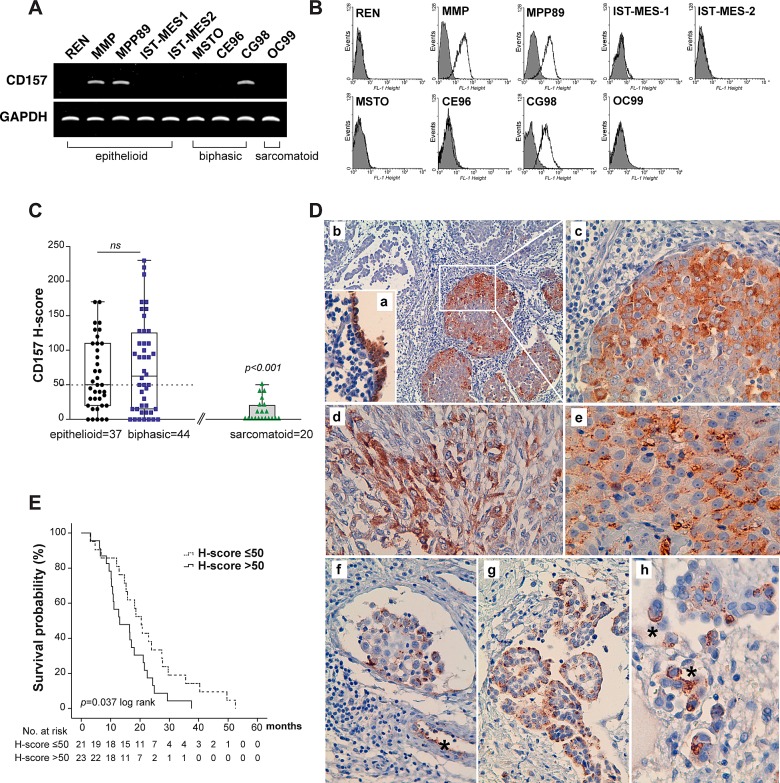
Expression of CD157 in MPM and correlation with patient outcome (A) RT-PCR analysis of CD157 in MPM cell lines. GAPDH was used as housekeeping gene. (B) CD157 expression in MPM cell lines by flow cytometry. Cells were stained with the RF3 anti-CD157 mAb (unshaded peaks) or isotype-matched mAb (shaded peaks). (C) Comparison of CD157 H-score in MPM specimens. Tumors were sorted by histotype and plotted against the CD157 H-score. Each data point corresponds to the CD157 H-score of an individual tumor. Boxes indicate the range (25^th^-75^th^ percentiles), whiskers indicate major and minor values; the horizontal line within the boxes indicates the median H-score of each group. The median H-score of 50 was used as the cut-off criterion for the epithelioid and biphasic MPM (dotted line). (D) Immunohistochemical staining of CD157 in MPM. (a) Expression of CD157 in peritumoral, non-malignant mesothelial cells (magnification ×200); (b) clonal expression of CD157 in tumor cell clusters in epithelioid MPM (×100); (c) computerized magnification of the white square in panel b highlighting granular cytoplasmic CD157 staining; (d) granular cytoplasmic CD157 staining in sarcomatoid component in biphasic MPM (×200); (e) CD157 membrane staining in epithelioid MPM (×200); (f) tumor infiltrate in a lymph vessel. (*) indicates CD157 staining of the endothelial lining of a blood vessel (×100). (g) CD157-positive cell clusters in epithelioid MPM (×100); (h) Membrane localization of CD157 with (*) strong apical staining (×400). E, Kaplan-Meier analysis of survival in 44 patients with biphasic MPM dichotomized for CD157 expression at the median H-score (= 50).

To explore the feasibility of studying CD157 in MPM, we first examined CD157 expression in nine human MPM cell lines. CD157 mRNA and protein expression was detected in two out of five epithelioid MPM cell lines (CD157-positive: MMP and MPP89; CD157-negative: REN, IST-MES-1 and IST-MES-2) and in one out of three biphasic MPM cell lines (CD157-positive: CG98; CD157-negative: MSTO and CE96). OC99 sarcomatoid MPM cell line was negative (Fig. 1A, B). On the same panel of MPM cell lines we investigated the expression of CD38, the other member of the NADase/ADP-ribosyl cyclase gene family. CD38 was detected in the IST-MES-1 and IST-MES-2 epithelioid MPM and in CE96 biphasic MPM cell lines (Supplementary Fig. S1).

We proceeded with a retrospective analysis of CD157 expression in 81 MPM surgical specimens (37 epithelioid and 44 biphasic; no specimens from sarcomatoid patients as surgery was not an option). Patient characteristics are reported in Supplementary Table S1. By immunohistochemistry, CD157 was detected in 85.2% of the specimens, evenly distributed among epithelioid (86.5%) and biphasic (84%) tumors. The CD157 H-score varied considerably among samples, ranging from 10 to 230 (median = 50), with no statistically significant difference between epithelioid and biphasic histotypes (*p* = 0.6654, Mann-Whitney U test). With the overall median H-score as cut-off, tumor specimens were divided into those with CD157 H-score ≤50 or H-score >50 (Fig. 1C).

Discrete subcellular patterns of CD157 localization were observed: cytoplasmic staining with diffuse granular or perinuclear spots was prevalent in 52.2% of surgical MPM tissues (Fig. 1D, panels b-d) while staining was mainly in the plasma membrane with apical localization in 47.8% of the specimens (Fig. 1D, panels e-h). Subcellular localization was independent of CD157 H-score and histotype (*p* = 0.116 and *p* = 0.821, respectively, Fisher's exact test). In biphasic MPM, both epithelioid and sarcomatoid components expressed CD157. Homogeneous CD157 staining was also detected in mesothelial cells adjacent to the tumor (Fig. 1D, panel a) and, as expected [9], in the endothelial lining of blood vessels (Fig. 1D, panel f).

CD157 expression was also evaluated in 20 consecutive thoracoscopic biopsies from patients with sarcomatoid MPM (Supplementary Table S2). Nine out of twenty (45%) specimens expressed CD157. The H-score ranged from 10 to 50, a statistically significant difference in distribution compared to the CD157 H-score observed in epithelioid and biphasic MPM (*p* < 0.001; Fig. 1C).

### CD157 expression correlates with clinical variables and survival in MPM

CD157 expression did not associate with sex, patient age at surgery, histology, asbestos history, disease stage or patient outcome when the 81 surgical MPM specimens were sorted according to the median CD157 H-score (Supplementary Table S3). The prognostic significance of the CD157 H-score and other clinical variables was estimated by univariate analysis for survival: tumor histology and advanced stage of disease correlated with a statistically significant increased risk of death (Table 1A).

**Table 1:** Univariate and multivariate analysis of survival

A						
		Univariate analysis		Multivariate analysis		
	No. of cases	Median survival (months)	HR (95% CI)	*p*	HR (95% CI)	*p*
Sex			0.917 (0.551-1.527)	0.74	0.969 (0.535-1.755)	0.917
Male	60	17.067				
Female	21	21.567				
Age at surgery, years			1.480 (0.931-2.353)	0.095	1.322 (0.821-2.130)	0.25
<55	33	20.767				
≥55	48	16.833				
Asbestose exposure			1.064 (0.680-1.664)	0.785	1.046 (0.640-1.710)	0.858
No	40	20.433				
Yes	41	16.433				
Histological type			1.724 (1.074-2.768)	0.022	1.536 (0.924-2.556)	0.098
Epithelioid	37	19.6				
Biphasic	44	16.533				
Disease stage, IMIG			2.144 (1.327-3.466)	0.001	2.073 (1.148-3.744)	0.016
I/II/III	52	22.167				
IV	29	11.967				
Surgical treatment			1.009(0.618-1.648)	0.972	0.963 (0.547-1.697)	0.897
EPP	56	16.533				
P/D	25	18.633				
Resection margin status			1.340 (0.855-2.100)	0.199	1.122 (0.584-2.158)	0.729
radical (R0)	37	22.467				
non radical (R1)	44	15.733				
Post-surgical therapy			1.217 (0.779-1.900)	0.388	1.416 (0.835-2.402)	0.196
No	40	18.167				
Yes	41	16.533				
CD157-H-score			0.966 (0.619-1.509)	0.881	1.054 (0.628-1.768)	0.842
≤50	41	18.167				
>50	40	16.533				

B						
Biphasic MPM=44		Univariate analysis		Multivariate analysis		
	No. of cases	Median survival (months)	HR (95% CI)	*p*	HR (95% CI)	*p*
Sex			1.381 (0.661-2.884)	0.388	1.304 (0.582-2.918)	0.519
Male	34	17.067				
Female	10	15.267				
Age at surgery, years			1.120 (0.601-2.087)	0.722	0.735 (0.318-1.699)	0.471

<55	17	18.000				
≥55	27	16.533				
Asbestose exposure			1.128 (0.617-2.063)	0.695	1.142 (0.562-2.322)	0.713
No	20	18.167				
Yes	24	15.733				
Disease stage, IMIG			1.990 (1.050-3.743)	0.03	1.500 (0.494-4.553)	0.474
II/III	27	20.767				
IV	17	12.900				
Surgical treatment			2.219 (1.081-4.556)	0.026	1.335 (0.474-3.760)	0.584
EPP	31	17.067				
P/D	13	15.267				
Resection margin status			1.609 (0.877-2.953)	0.121	1.383 (0.365-5.235)	0.633
radical (R0)	21	20.433				
non radical (R1)	23	15.267				
Post-surgical therapy			0.894 (0.488-1.639)	0.717	0.932 (0.380-2.284)	0.877
No	19	18.167				
Yes	25	16.533				
CD157-H-score			1.933 (1.029-3.631)	0.037	2.433 (1.120-5.284)	0.025
≤50	21	20.433				
>50	23	13.067				

CI = confidence interval; HR= hazard ratio

*p* values were calculated using two-sided log-rank test for univariate analysis and two-sided Wald test for multivariate analysis.

EPP, extrapleural pneumonectomy; P/D, pleurectomy/decortication

Because epithelioid and biphasic MPM have different prognoses, the correlation between survival and CD157 H-score or other clinical variables was analysed separately in the two histotypes. In epithelioid MPM, univariate analysis indicated that only early tumor stages were associated with longer survival times (early *versus* late stages, HR = 2.260, 95% CI = 1.061 to 4.814, *p* = 0.035); there was no correlation between survival and CD157 H-score (data not shown). In biphasic MPM, in addition to advanced stage of disease and pleurectomy/decortication, the CD157 H-score >50 also correlated with poor prognosis (Table 1B). By Kaplan-Meier analysis, median survival was 13.067 months in patients with biphasic MPM and CD157 H-score >50 (95% CI = 4.771 to 21.362); with CD157 H-score ≤50, the median survival was 20.433 months (95% CI = 16.546 to 24.321; log-rank test *p* = 0.037) (Fig. 1E). The multivariable Cox proportional hazard model applied to biphasic MPM confirmed that CD157 H-score was an independent predictor of survival (Table 1B).

In biphasic MPM, subcellular CD157 localization also correlated with statistically significant differences in survival, with membrane localization associated with the worst prognosis (HR = 2.031, 95% CI = 1.022 to 4.038). Kaplan-Meier analysis stratified by CD157 subcellular localization demonstrated that median OS was 13.067 months (95% CI = 7.509 to 18.624) for biphasic MPM patients with membrane CD157 and 18.633 months (95% CI = 13.301 to 23.966; log-rank test *p* = 0.039) for patients with cytoplasmic CD157 (Supplementary Fig. S2A). Biphasic MPM patients with CD157 H-score >50 and membrane CD157 had the worst prognosis: median OS was 10.9 months (95% CI = 9.102 to 12.698) *versus* 16.533 months for patients with biphasic MPM, CD157 H-score >50 and cytoplasmic CD157 (95% CI = 13.874 to 19.193; log-rank test *p* = 0.044) (Supplementary Fig. S2B).

### Linking CD157 expression to mesothelioma cell growth and tumorigenicity

**Figure 2:**
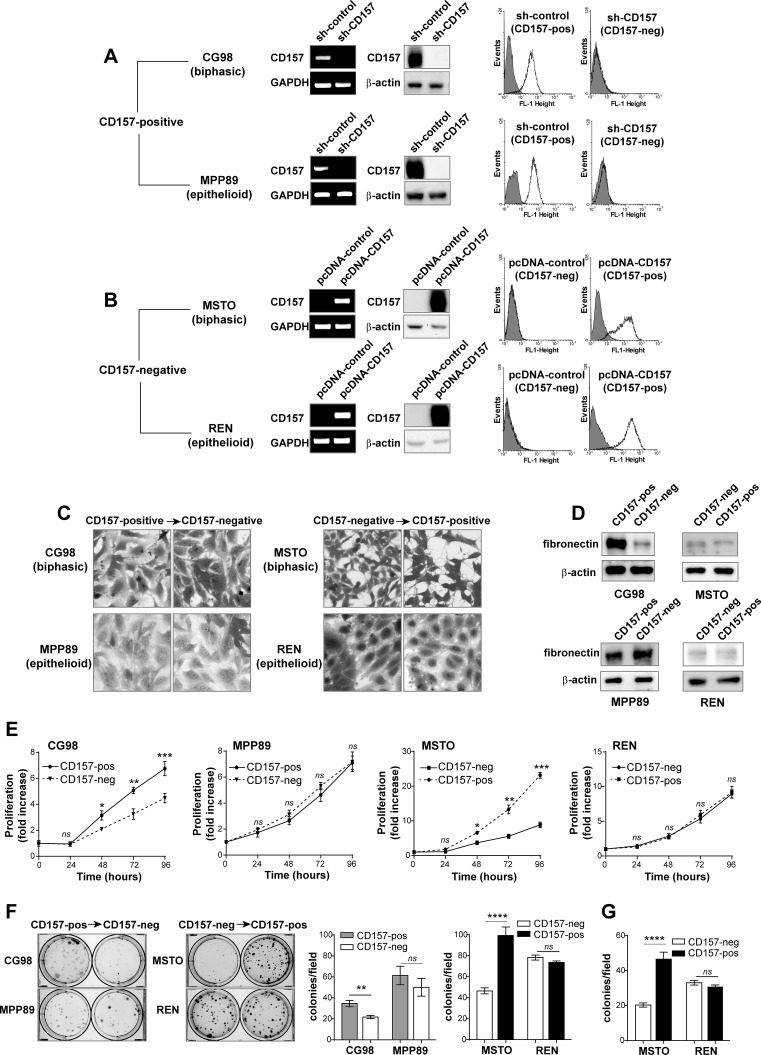
CD157 influences MPM cell morphology and growth (A) CG98 and MPP89 cells were stably transduced with a CD157-specific shRNA (sh-CD157) or a scrambled shRNA (sh-control). (B) MSTO and REN cells were stably transfected with the pcDNA3.1 expression vector containing full-length CD157 cDNA (pcDNA-CD157), or with the empty vector (pcDNA-control). CD157 mRNA and protein expression were analysed by RT-PCR, western blot and flow cytometry. GAPDH, β-actin and an isotype matched mAb (gray shaded peaks) were used as controls, respectively. (C) Light microscopy images showing the morphological features of cells with or without CD157 stained with crystal violet and reproduced in black and white (magnification ×10). (D) Western blot for fibronectin. (E) MTT viability assay of MPM cells. Results represent the mean ± SEM of three independent experiments performed in quadruplicate. (F) Colony formation assay of MPM cells. Colonies were stained with crystal violet and reproduced in black and white. (G) Clonogenic assay in soft agar. Columns represent the average number of colonies/10 fields from three independent experiments ± SEM. ****p < 0.0001, ***p < 0.001,**p < 0.01,*p < 0.05, *ns* = not significant, two-tailed unpaired t test.

The finding that CD157 expression correlated with poor prognosis in patients with biphasic MPM triggered an investigation into the influence of CD157 on tumor cell behavior *in vitro*. Based on the analysis of CD157 expression in MPM cell lines reported above, we selected CD157-positive (biphasic CG98 and epithelioid MPP89) cell lines for gene knockdown to investigate the effects of loss of CD157 expression. In brief, from the parental CD157-positive cell lines, we generated stable CD157 knockdown versions (with CD157-specific shRNA) and controls that conserved endogenous expression of CD157 (with control shRNA). In a complementary approach, we selected CD157-negative (biphasic MSTO and epithelioid REN) cell lines and transfected them to assess the effects of gain of CD157 expression. From the parental CD157-negative cell lines, we generated CD157-positive versions by stable CD157 cDNA transfection (and empty vector control cell lines that remained constitutively CD157-negative). The CD157 status of the eight genetically engineered cell lines was assessed by RT-PCR, western blot and flow cytometry (Fig. 2A, B). Endogenous and exogenous expression levels of CD157 were comparable whereas CD157 shRNA-mediated knockdown effectively abolished expression of CD157 (Fig. 2A, B).

We first evaluated the effects induced by loss or gain of CD157 expression on cell morphology, growth and tumorigenicity. By light microscopy, we observed that CD157 expression affected cell morphology in biphasic, but not epithelioid, cell lines. Morphology was more spindle-like with reduced cell spreading in the biphasic CD157-positive (endogenous CG98 or exogenous MSTO) cell lines compared to their CD157-negative (knockdown CG98 or constitutive MSTO) counterparts (Fig. 2C). This spindle-shaped morphology was associated with abundant expression of fibronectin, especially in CD157-positive CG98 cells. However, in the CG98 CD157 knockdown cells, there was a striking reduction in fibronectin expression (Fig. 2D). Contrasting with the impact of CD157 in biphasic MPM cell lines, epithelioid cell line morphology and fibronectin expression remained essentially unchanged by modulation of CD157 (Fig. 2C, D).

In cell proliferation assays, CD157 expression affected the biphasic, but not epithelioid, MPM cell lines: loss of CD157 significantly reduced the growth of CG98 but not of MPP89 cells compared to their CD157-positive controls, whereas forced expression of CD157 enhanced the growth of MSTO but not those of REN cells (Fig. 2E). As a further marker of cell growth, plating efficiency was examined: CD157 knockdown reduced the number and size of colonies in CG98 cells whereas CD157 expression significantly increased plating efficiency in MSTO cells. MPP89 and REN cells with or without CD157 showed similar number of colonies of comparable size (Fig. 2F). By soft agar assay, a conventional *in vitro* measure of cell tumorigenicity, exogenous expression of CD157 led to a significant increase in number of colonies in MSTO cells, but not in REN cells (Fig. 2G). CG98 and MPP89 cells were unable to grow in the absence of anchorage (regardless of the expression of CD157), hampering their use in soft agar assays.

### Linking the expression of CD157 to MPM cell migration and invasiveness

**Figure 3:**
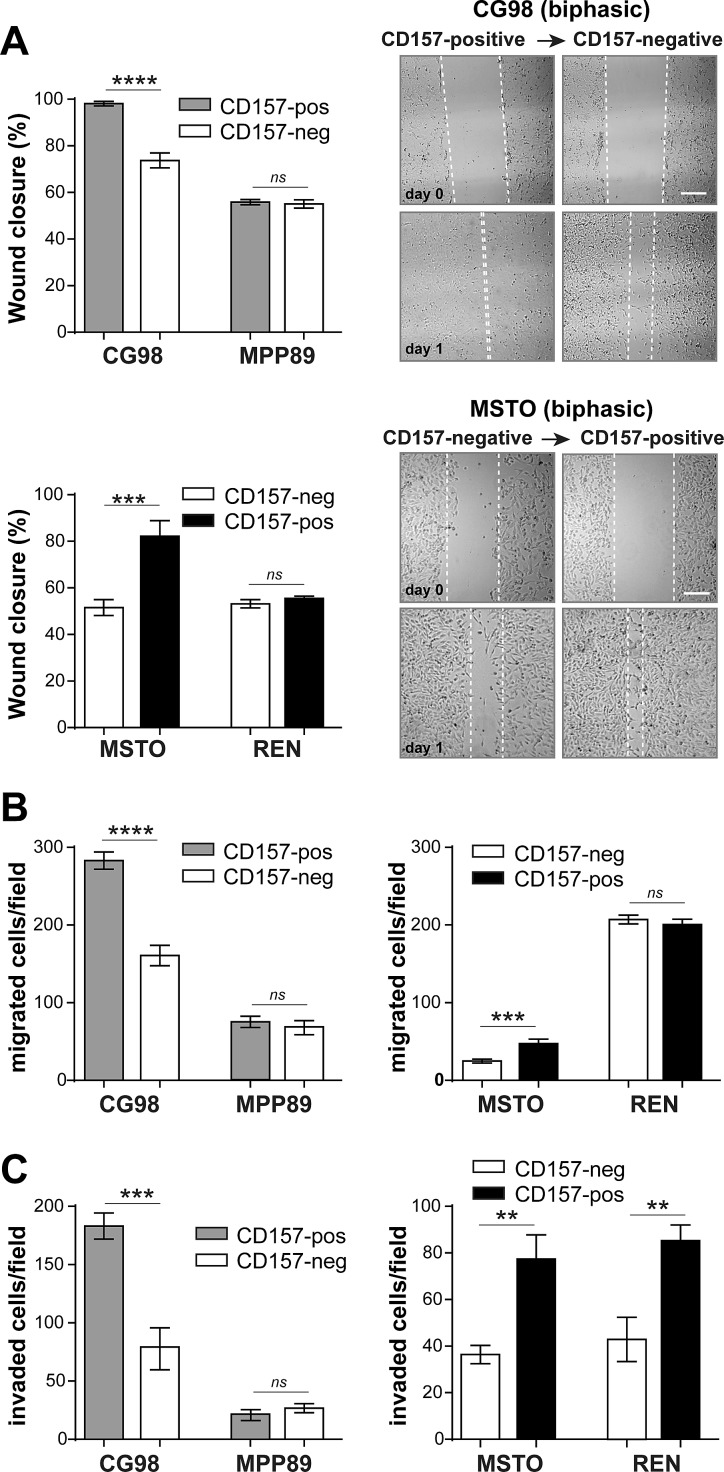
CD157 increases MPM cell motility and invasiveness (A) Wound-healing assay to assess CD157 and cell motility as quantified by percent wound closure over 24 hours (scale bar: 200 μM). (B) Transwell® assay to assess CD157 on cell migration toward 10% FBS. (C) Matrigel assay to assess CD157 on cell invasion. Migrated or invading cells were stained and counted in ten microscopic fields at ×100 magnification. Results represent the mean ± SEM of three independent experiments. ****p < 0.0001, ***p < 0.001, **p < 0.01, *p < 0.05, *ns* = not significant, two-tailed unpaired t test.

The propensity of MPM to spread by invasive growth along the pleura is a major determinant of tumor outcome. We investigated the relevance of CD157 expression in cell motility and invasiveness using three conventional assays of tumor aggressiveness. First, in wound healing scratch assays, CD157 knockdown in CG98 cells led to a marked reduction (~25%) in wound closure at 24 hours compared to CD157-positive CG98 cells. Conversely, gain of CD157 in MSTO cells increased motility. Gain or loss of CD157 did not affect motility in the epithelioid MPP89 and REN cells (Fig. 3A). Second, by directional migration assays (towards 10% FBS used as the chemoattractant) loss or gain of CD157 expression altered migration in CG98 and MSTO biphasic MPM but not in MPP89 and REN epithelioid MPM (Fig. 3B). Third, we assessed the effect of CD157 on MPM cell invasiveness by Matrigel invasion assays. CD157 knockdown in biphasic CG98 decreased cell invasion compared to the CD157-positive counterpart. MPP89 cells were poorly invasive independently of CD157 expression. Instead, gain of CD157 in biphasic MSTO and epithelioid REN cells markedly increased their invasive potential with respect to their CD157-negative controls (Fig. 3C).

### CD157 modulates the activation of mTOR

**Figure 4:**
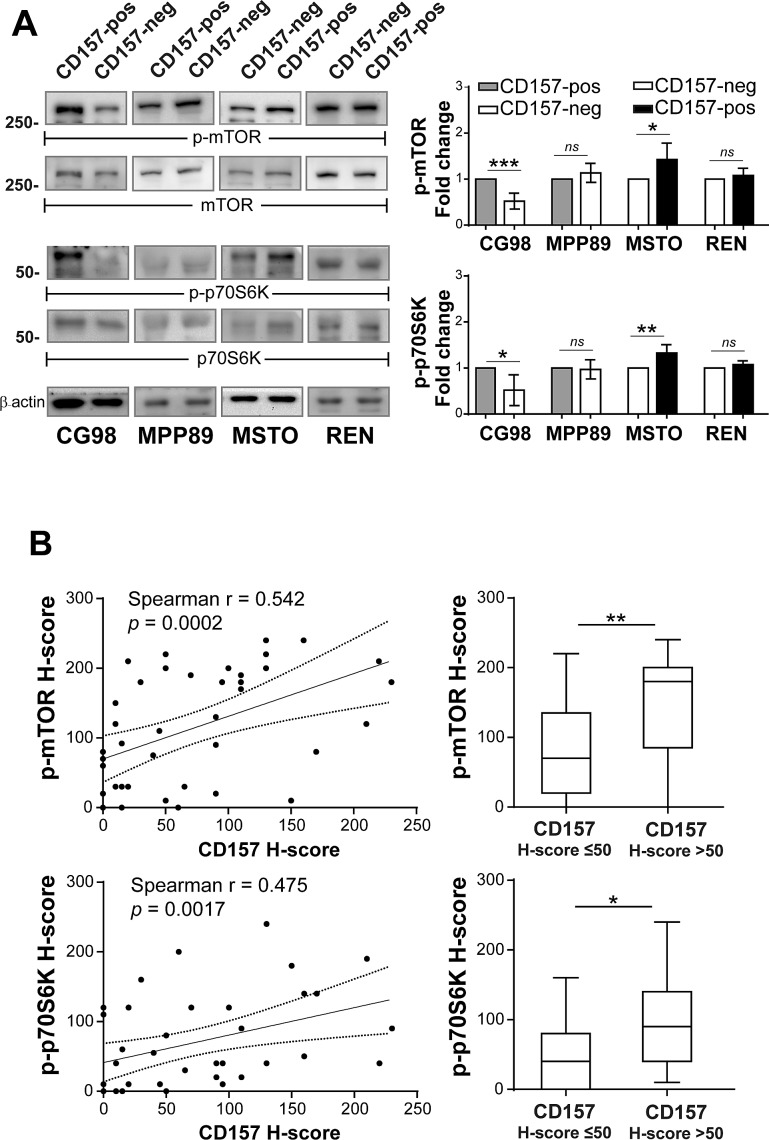
Linking CD157 expression with mTOR activation in biphasic MPM (A) Western blot of phosphorylated mTOR and p70S6K proteins in CD157-positive and negative cells grown in medium with 10% FBS for 24 hours. β-actin was used as loading control. Results of densitometric analysis are expressed as fold changes compared to each corresponding control and are the means ± SD of three independent experiments. (B) CD157 H-score correlates with the p-mTOR and p-p70S6K H-scorers in biphasic MPM specimens (3 missing due to lack of material, left panels). Distribution of p-mTOR and p-p70S6K H-scores (right panels) confirmed that tumors with CD157 H-score >50 had higher median p-mTOR and p70S6K H-score than tumor with CD157 H-score ≤50 (*p* = 0.0085 and *p* = 0.0125, respectively). Boxes indicate the range (25^th^-75^th^ percentiles), whiskers indicate major and minor values, and the horizontal line within the boxes indicates the median H-score of each group.

The PI3K/Akt/mTOR pathway plays a key role in the progression of a number of solid tumors, including mesothelioma (12-15). We examined the activation of mTOR and its downstream effector p70 ribosomal S6 kinase1 (p70S6K) in CD157-positive and negative MPM cells cultured in standard conditions. By western blot, mTOR phosphorylation was evident in all the MPM cell lines analysed; however, phospho-mTOR (p-mTOR) and phospho-p70S6K (p-p70S6K) were more elevated in CG98 and MSTO biphasic cell lines expressing CD157 (endogenous or exogenous) than in their CD157-negative counterparts. Gain or loss of CD157 expression in MPP89 and REN epithelioid cell lines did not significantly perturb the phosphorylation status of mTOR and p70S6K (Fig. 4A).

Following this result, linking CD157 expression in biphasic MPM lines with increased mTOR phosphorylation *in vitro*, we assessed staining and calculated the H-score for p-mTOR and p-p70S6K in the same MPM surgical specimens previously used to detect CD157 expression. In biphasic MPM tissue samples, there was a positive linear correlation between the H-score of CD157 and both those of p-mTOR and of p-p70S6K (Fig. 4B, left panels). The analysis of distribution of p-mTOR and p-p70S6K H-scores demonstrated that biphasic MPM with CD157 H-score >50 had significantly higher median H-score of p-mTOR and p-p70S6K than those with CD157 H-score ≤50 (Fig. 4B, right panels). In epithelioid MPM no correlation was found between CD157 and p-mTOR H-scores (r = 0.2826, *p* = 0.0902 Spearman's correlation test) or p-p70S6K H-scores (r = 0.1279, *p* = 0.4506, Spearman's correlation) (n = 37, data not shown).

### CD157 modulates responsiveness to chemotherapy through mTOR activation

**Figure 5:**
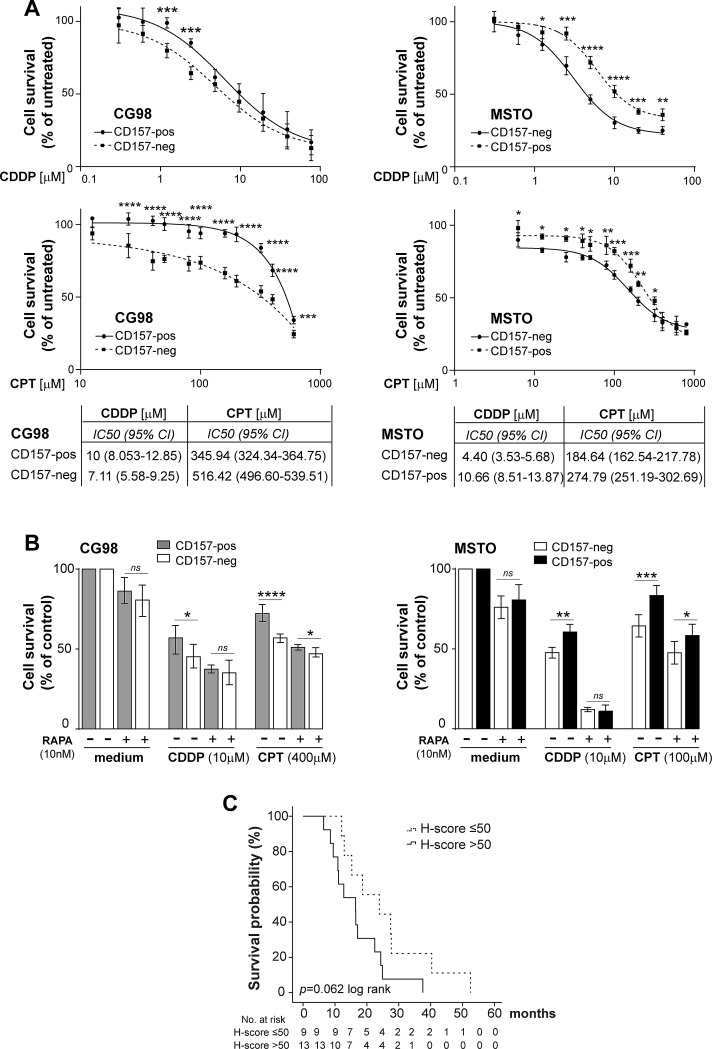
CD157 modulates responsiveness to chemotherapy in biphasic MPM (A) Drug sensitivity assays showing the viability of CD157-positive and negative cells treated with increasing concentrations of CDDP (top panels) or CPT (bottom panels) for 48 hours. Absorbance was read and each data was normalized to the respective untreated control and plotted. Results represent the mean ± SEM of at least three experiments performed in quadruplicate. Each IC50 is indicated. (B) Effect of rapamycin on CG98 and MSTO cells sensitivity to CDDP and CTP. Cells were exposed to 10 nM rapamycin for 8 hours before treatment with CDDP, CPT or vehicle for 48 hours. Data are the mean ± SD of one representative experiment of three performed in quadruplicate. ****p < 0.0001, ***p < 0.001, **p < 0.01, *p < 0.05, *ns* = not significant. (C) Patients with biphasic MPM who received platinum-based chemotherapy were subdivided according to median CD157 H-score. Kaplan-Meier analysis shows that patients with CD157 H-score >50 (solid line) had a shorter (albeit not statistically significant, log-rank test *p* = 0.062) survival than patients with H-score ≤50 (dashed line).

The activation of the mTOR/p70S6K axis is also implicated in resistance to chemotherapy, including platinum compounds widely used to treat MPM [16, 17]. We used our CD157-positive and negative MPM cell line models to assess the cytotoxic effect of increasing concentrations of cisplatin (CDDP) or carboplatin (CPT), and to establish their cytotoxic potency (IC50). Biphasic cell lines expressing CD157 were significantly more resistant to both CDDP and CPT than their CD157-negative counterparts. In CG98, although CD157-positive and negative cells had similar IC50, the CD157-positive cells showed greater resistance to treatment for 48 hours with low-dose (1.25-2.5 μM) CDDP. Likewise, CD157-positive CG98 was more resistant to CPT treatment compared to CG98 CD157-negative cells (Fig. 5A, left panels). MSTO CD157-positive cells were significantly more resistant to both CDDP and CPT treatment, and showed a markedly increased IC50 value compared to MSTO CD157-negative cells (Fig. 5A, right panels). In MPP89 and REN epithelioid cell lines, gain or loss of CD157 did not influence their sensitivity to CDDP or CPT (Supplementary Fig. S3).

To investigate if the link between CD157 expression and platinum sensitivity in biphasic MPM was associated with mTOR activation, CD157-positive and -negative CG98 and MSTO cells were exposed to 10 nM rapamycin (mTOR inhibitor [18]) for 8 hours before treating with CDDP or CPT at concentration close to each IC50 values. Rapamycin alone moderately reduced viability in all cell lines. Exposure to rapamycin then to CDDP or CPT enhanced cytotoxicity compared to treatment with either CDDP or CPT alone. Notably, when pretreated with rapamycin, biphasic CG98 and MSTO CD157-positive cell lines showed significant recovery of sensitivity to CDDP and CPT (Fig. 5B).

Finally, we explored the relationship between CD157 expression and response to platinum-based chemotherapy *in vivo*, defined in terms of survival advantage. The influence of CD157 expression on survival was evaluated in 41 patients in our cohort, treated postoperatively with platinum-based chemotherapy (22/44 patients with biphasic and 19/37 with epithelioid MPM). By univariate analysis, chemotherapy did not predict survival benefit for patients with either biphasic (log-rank test *p* = 0.419) or epithelioid (log-rank test *p* = 0.114) MPM. However, when chemotherapy-treated or untreated biphasic MPM patients were grouped according to CD157 H-score, the group with chemotherapy and CD157 H-score >50 showed a clear trend towards shorter survival than the group with chemotherapy and CD157 H-score ≤50, although the difference was not statistically significant (log-rank test *p* = 0.062) (Fig. 5C). In contrast, the 40 patients who did not receive postoperative chemotherapy showed no correlation between CD157 H-score and survival (log-rank test *p* = 0.178, data not shown). No prognostic correlation was observed in patients with epithelioid MPM among chemotherapy response, CD157 H-score and survival (data not shown). These data suggest that CD157 could be a promising candidate as a predictive marker of response to platinum-based therapy in biphasic MPM patients.

## DISCUSSION

The increasing worldwide incidence of MPM poses many urgent challenges. Among these, a better understanding of the molecular mechanisms driving MPM progression is a priority as it will ultimately translate into better diagnosis and treatment.

In this paper, we report that CD157/BST1 is expressed in the most common types of MPM, and in several MPM cell lines. High CD157 expression was found consistently associated with exacerbated tumor aggressiveness in the biphasic histotype both *in vitro* and *in vivo*. Indeed, i) in the CD157-negative/CD157-positive biphasic MPM cell line models, CD157 expression correlated with enhanced cell growth, migration, invasion and resistance to platinum-based chemotherapy; ii) in patients with biphasic MPM, high CD157 H-scores correlated with shorter survival with respect to patients with low CD157 H-score. The independent prognostic value of CD157 in biphasic MPM was further confirmed by multivariate survival analysis.

Our preceding studies have clearly demonstrated that CD157 can confer malignant and invasive traits to epithelial ovarian cancer by promoting mesenchymal differentiation [10]. More recent studies have shown that CD157 regulates cell adhesion to the extracellular matrix (ECM) [19] and binds selected ECM proteins, including fibronectin [20] which has a central role in mesenchymal differentiation [21]. This data indicates that CD157 is involved in EMT and suggests that the CD157/ECM interplay in the tumor microenvironment could be critical in determining the aggressiveness of CD157-positive tumors by facilitating the EMT differentiation program ongoing in biphasic MPM [5, 22]. This is supported by the observation that the expression of CD157 emphasized some distinctive mesenchymal properties of biphasic CG98 and MSTO MPM cell lines, such as spindle-shape morphology, motility, invasiveness and resistance to chemotherapy [23]. Apparently, CD157 expression did not impinge upon MPP89 and REN epithelioid cell morphology, motility and drug resistance. However, when cultured in the presence of a surrogate ECM (such as Matrigel) that generates a more permissive environment, CD157-positive epithelioid REN cells showed enhanced invasiveness with respect to CD157-negative cells. This suggests that CD157 relies on specific microenvironmental cues to accomplish its function. In MPM, each histotype produces different growth factors and ECM proteins, influencing the composition of the tumor microenvironment and the proclivity of each tumor to spread and disseminate [22, 24]. It is tempting to envision that *in vivo* the local auto/paracrine secretion of different ECM components affects the surrounding tumor microenvironment thereby determining the pro-tumorigenic functions of CD157 in patients with MPM. However, whether the CD157-driven pro-tumorigenic effect is exclusively due to its interaction with the ECM or whether it is dependent on other mechanisms implemented in the tumor niche (such as, CD157's involvement in the cancer cells/stroma interaction) remains to be established.

The acquisition of EMT and gain of stem-like features are strictly interconnected and contribute to tumor growth and dissemination. Since CD157 is a marker of stem cell both in human [25] and mouse [26, 27], the hypothesis that CD157 serves as a point of convergence in conferring mesenchymal and stem-like differentiation to mesothelioma cells is intriguing and deserve further investigation.

In MPM tumor tissues, CD157 expression was heterogeneous both in terms of H-score and subcellular localization, a feature common to many other markers in solid tumors [28, 29]. At present, we do not know how high *versus* low and membrane *versus* cytoplasm patterns of CD157 expression affect its role in MPM. However, patients with high and membrane-localized CD157 expression had a worse prognosis than patients with cytoplasmic localization, further corroborating the notion that the interaction with currently unidentified components in the tumor microenvironment is critical for CD157 to exert its functions.

Increasing evidence indicates that the Akt/mTOR pathway is frequently activated in MPM [30] and plays a key role in tumor aggressiveness and chemoresistance [15, 31, 32]. However, in MPM, the molecular mechanism(s) leading to activation of mTOR and its interaction with other biomarkers are largely unknown. The interplay between CD157 and the mTOR pathway recently demonstrated in mice [33] and the role of CD157 in MPM aggressiveness prompted us to investigate this pathway. Several lines of evidence suggest that CD157 exerts its pro-tumorigenic effects through the activation of mTOR. First, consistently greater phosphorylation of mTOR and its downstream effector p70S6K was found in CD157-positive *versus* CD157-negative biphasic (but not epithelioid) MPM cell lines. Second, a positive correlation emerged between the expression of CD157 and the extent of mTOR and p70S6K phosphorylation in primary tumors from patients with biphasic MPM, thus strengthening the existence of a relationship between CD157 expression and activation of mTOR *in vivo*.

Activation of mTOR has an established role in the generation of chemotherapy resistance in several tumors [34, 35]. Consistently, both CD157-positive CG98 and MSTO biphasic cell lines showed reduced sensitivity to cisplatin and carboplatin treatment compared to the corresponding CD157-negative cells. Although further studies are required before drawing definitive conclusions, clinical data indicated that patients with biphasic MPM and high CD157 H-score receiving platinum-based therapy had shorter survival than patients with low CD157 H-score. Finally, treatment with rapamycin (a prototypic mTOR inhibitor) [36] restored sensitivity in cisplatin- and carboplatin-resistant CD157-positive CG98 and MSTO cells, confirming that CD157-mediated platinum resistance at least partly relies on the activation of mTOR. Overall, our *in vitro* and *in vivo* studies point to CD157 as a promising marker of efficacy for treatment with platinum compounds in patients with biphasic MPM. So far, the availability of dependable predictive markers of response to therapy is poor, among these CD26 is emerging as a promising candidate since its expression was found associated with better response to chemotherapy [37]. As mesothelioma represents a great challenge to clinicians and researchers due to its poor prognosis and marked resistance to current therapies [4, 38], finding markers that will identify group of patients most likely to benefit from a specific chemotherapy regimen is an important goal.

Collectively, our data highlight a pivotal role of CD157 in multiple aspects of MPM progression and suggest that CD157 has clinical potential as a marker for stratifying patients with biphasic MPM into different prognostic groups. Moreover, CD157 may identify patients with highly aggressive MPM which might benefit from a particular chemotherapeutic approach that may include mTOR inhibitors.

## MATERIALS AND METHODS

### Antibodies and reagents

RF3 anti-CD157 monoclonal antibody (mAb) (IgG1, MBL International) and anti-fibronectin (Sigma-Aldrich) were used. Horseradish peroxidase (HRP)-conjugated goat-anti-rabbit, HRP-goat-anti-mouse polyclonal IgG and HRP-anti-β-actin (sc-1615) antibodies were from Santa Cruz Biotechnologies. Anti-mTOR (L24D4, #4517), anti-phospho-mTOR (Ser2448/49F9, #2976), anti-p70S6K (49D7, #2708) and anti-phospho-p70S6K (Thr389/1A5, #9206) antibodies were from Cell Signalling Technology. FITC-labeled F(ab′)_2_ fragments from goat antibodies to mouse IgG were from Jackson ImmunoResearch. Original stock solutions of cisplatin (cis-diammine-dichloroplatinum; Pfizer) (1 mg/ml) and carboplatin (Platinum, diammine[1,1-cyclobutanedicarboxylato(2-)-O,O'], Teva Pharma Italia) (10 mg/ml) were stored at -20°C and freshly dissolved in culture medium at time of use.

### Patients and tissue samples

Tissue sections were retrospectively obtained from 81 consecutive specimens (37 epithelioid; 44 biphasic) from patients operated for MPM between 1998 and 2005 at the San Giovanni Hospital (Torino, Italy) [39, 40]. Because of its surgical origin, this cohort did not reflect the expected prevalence of the epithelioid histological type. Patient characteristics are reported in Suppl. Table S1. Tumor stage was determined according to the International Mesothelioma Interest Group (IMIG) staging system. Occupational exposure to asbestos was documented in 41 patients. Median overall survival (OS) was 18 months. We obtained 20 consecutive thoracoscopic biopsies of sarcomatoid MPM diagnosed between 2004 and 2010 at the San Luigi Hospital (Orbassano, Italy). A pathologist blinded to the purpose of the study anonymized samples and clinical data. The study was approved by the local Institutional Review Board.

### Immunohistochemistry

Five *μ*m deparaffinized sections from formalin-fixed MPM samples were analysed using RF3 (anti-CD157), 49F9 (anti-phospho-mTOR) and 1A5 (anti-phospho-p70S6K) mAbs, as previously described by our groups [11, 41]. All samples were evaluated using a semi-quantitative histological score (H-score) which took into account both the percentage of positive tumor cells within the whole section and the intensity of immunostaining. The H-score was calculated by summing percentage of cells stained at each intensity level (0–100) multiplied by the weighted intensity (0, none; 1, weak; 2, moderate; 3, intense), generating for each tumor a semi-quantitative score ranging from 0 to 300.

### Cell lines, transfections and transductions

The human MPM cell lines MSTO-211H (designated MSTO) [42], MPP89, IST-MES-1 and IST-MES-2 [43] were purchased from Interlab Cell Line Collection (Genova, Italy), REN [44] was kindly provided by L. Moro (University of Novara, Italy) who received the cells from S. Albelda (University of Pennsylvania, Philadelphia, USA); CE96, OC99, CG98 and MMP cell lines were obtained from the mesothelioma bio bank, Pathology Unit, City Hospital of Alessandria (Italy) [45]. Cells were cultured as recommended by each provider. All cell lines were thawed from early-passage frozen stocks and were passaged less than twenty times prior to use. Cell lines were regularly examined for morphology; *Mycoplasma* contamination was excluded using a PCR-based assay. CD157 knockdown was obtained by lentiviral delivery of pLV-puro plasmid (Biosettia) encoding a short hairpin RNA (shRNA) targeting the 3′ UTR of BST1 mRNA, as described (10). Scrambled shRNA controls were always included. CD157 expression was obtained by transfection with the pcDNA3.1 eukaryotic expression vector (Invitrogen) containing full-length CD157 cDNA (GenBank NM_004334). Control was empty vector, as described [11].

### RNA extraction and reverse transcriptase-PCR

Total RNA (2 μg) extracted from 70-80% confluent cell cultures using TRI Reagent® (Sigma Aldrich) was reverse-transcribed with the M-MLV Reverse Transcriptase (Thermo Fisher Scientific) and Oligo-dT primers. cDNA was amplified using KAPA2G Fast HotStart DNA Polymerase (Kapa Biosystems, Wilmington, MA, USA). Each cycle consisted of denaturation at 95°C for 10 s, annealing for 10 s and extension at 72°C for 1 s. The primers used were as follow: CD157, 5′-CCAAAGTTCCCCGATGGCGGCC-3′ (forward) and 5′-GGTTAGAGCAACACAGTTTCC-3′ (reverse), GAPDH, 5′-GAGTCAACGGATTTGGTCGT-3′ (forward) and 5′-TTGATTTTGGAGGGATCTCG-3′ (reverse). PCR products were analyzed by electrophoresis on 1% agarose gel stained with Midori-green (Nippon Genetics Europe, Dueren Germany).

### Flow cytometry

Engineered MPM cells were incubated with RF3 mAb (5 μg/ml) for 30 min at 4°C followed by F(ab′)_2_-GαMIg-FITC (5 μg/ml) for 30 min at 4°C. Background mAb binding was estimated by staining an identical cell population with an isotype-matched irrelevant control mAb. Fluorescence was analyzed using a FACSCanto flow cytometer (BD Biosciences). Ten thousand cells were considered for each analysis.

### Cells proliferation assay

Cells (3-5×10^3^/well) were seeded in 96-well plates in culture medium supplemented with 10% FBS for the indicated time. At the end of the experiment, MTT (5 mg/ml) was added to each well for 1 h at 37°C, then, the reaction was stopped by adding DMSO (Sigma-Aldrich) and cell proliferation evaluated. Absorbance was read at 570 nm with reference at 680 nm, using a microplate reader (Bio Rad). At each indicated time, fold increase of the optical density (OD) compared to time zero was plotted for each cell line and compared in CD157-positive *versus* CD157-negative cells at each time points using a Student's t test.

### Colony forming assay

Plating efficiency, a measurement of the number of colonies originating from single cells, was used to determine the clonogenic ability of MPM cells. Briefly, cells (500/well) were seeded in 6-well plates and maintained in culture medium with 10% FBS for 2 weeks, then washed, fixed with methanol for 30 min, stained with 0.2% crystal violet (Sigma-Aldrich) and counted using an IX70 inverted microscope equipped with a UC30 camera and the CellF analysis software (Olympus Biosystems).

### Soft agar assay

Cells (3×10^3^) were suspended in 1.5 ml of 0.45% low melting agar-containing RPMI with 10% FBS and then overlaid on a 0.9% agar base layer in 6-well culture plates. One ml of RPMI with 10% FBS was added on the top. After 2 weeks incubation at 37°C and 5% CO_2_, colonies were stained with 0.2% crystal violet, visualized under an inverted microscope and those with >50 cells were counted as described above.

### Wound healing assay

Cells were seeded in 24-well plates until confluence and a scratch was then made across the monolayer. Migration efficiency, expressed in term of percentage of wound closure, was calculated by measuring 20 randomly chosen distances across the wound at time 0 and 24 h, divided by the mean distance measured at time zero. Images of the wounded area were recorded using an Olympus Biosystems Microscope IX70, equipped with an UC30 camera and CellF software, at the indicated times.

### Chemotaxis and invasion assay

The directional migration of MPM cells was assayed using 24-Transwell® plates (BD Bioscence). For invasion assay, filters (8 μm pore size) were pre-coated with 30 μg/cm^2^ of Matrigel (BD Biosciences). Cells (1×10^5^) in serum-free medium were added to the top chamber and RPMI with 10% FBS was put in the lower compartment to stimulate cell migration or invasion. After 16 to 24 h, migrated cells on the bottom surface of the filters were fixed in methanol and stained with hematoxylin and eosin. The number of cells in 10 high-power fields (HPF, ×100) for each triplicate wells was counted and the average number was determined.

### Western-blot assay

Confluent MPM cells in culture were washed with PBS and cell extracts were prepared in RIPA buffer (Millipore) supplemented with 1 mM Na_3_VO_4_, 5 mM NaF, 50 μg/ml aprotinin and leupeptin (Sigma-Aldrich). Equal amounts of proteins were separated by SDS-PAGE, transferred to polyvinylidene difluoride (PVDF) membrane, and immunoblotted with the indicated antibodies. Images were captured with a ChemiDoc™ XRS+ System and densitometry analysis was performed with Image Lab™ Software (Bio Rad).

### Drug sensitivity assay

Cells were seeded in 96-wells plates in medium with 10% FBS for 16 h before treatment, to allow attachment without cell-doubling. Cells were starved overnight, then treated with the indicated concentrations of cisplatin or carboplatin for 48 h. Cell viability was determined by MTT assay by comparing the OD value before and after treatment and normalizing the data to controls (untreated cells = 100%). IC50 was defined as the concentration causing 50% reduction in OD value relative to the untreated control, and it was determined by non-linear regression and a four-parameter logistic model with log-transformed data. In selected experiments, after starvation, cells were treated for 8 h with 10 nM rapamycin (Cell Signaling Technology) alone or followed by the addition of cisplatin or carboplatin at the indicated concentrations. Cell viability was assessed at 48 h.

### Statistical analysis

Results of *in vitro* experiments are shown as means ± SEM, unless otherwise indicated. Comparisons among groups were performed with one-way ANOVA models; differences between two groups were analysed using two tailed t tests. The association between CD157 tumor expression and clinical data was analysed using the χ^2^ test or Fisher exact test. Overall survival (OS) was defined as the time interval from the date of surgery to the date of death for disease, or to the date of last follow-up for still alive patients. OS was analysed using Kaplan–Meier curves; differences in survival between groups of patients were assessed using the log-rank test. Univariate and multivariable survival analyses were performed for all available clinical-pathologic variables, including CD157 H-score, using the Cox proportional hazards regression model. Covariates that were not statistically significantly associated with survival were not removed from the model (complete model). Crude and adjusted hazard ratios (HR) with relative 95% confidence interval (CI) are shown. Spearman's test was used for correlation analysis between two factors. SPSS Statistics 20 and GraphPad Prism 6 were used for all statistical analyses. All statistical tests were two-sided. *p* < 0.05 was considered statistically significant.

## SUPPLEMENTARY MATERIAL FIGURES AND TABLES


