# Supplementary material for: CD157 enhances malignant pleural mesothelioma aggressiveness and predicts poor clinical outcome
Source: Oncotarget. 2014 Jul 8;5(15):6191–205. doi: 10.18632/oncotarget.2186 (PMC4171622; doi:10.18632/oncotarget.2186)
Supplement: Supplementary file 1 [file oncotarget-05-6191-s001.pdf]

CD157 enhances malignant pleural mesothelioma aggressiveness and predicts poor clinical outcome

Supplementary Material

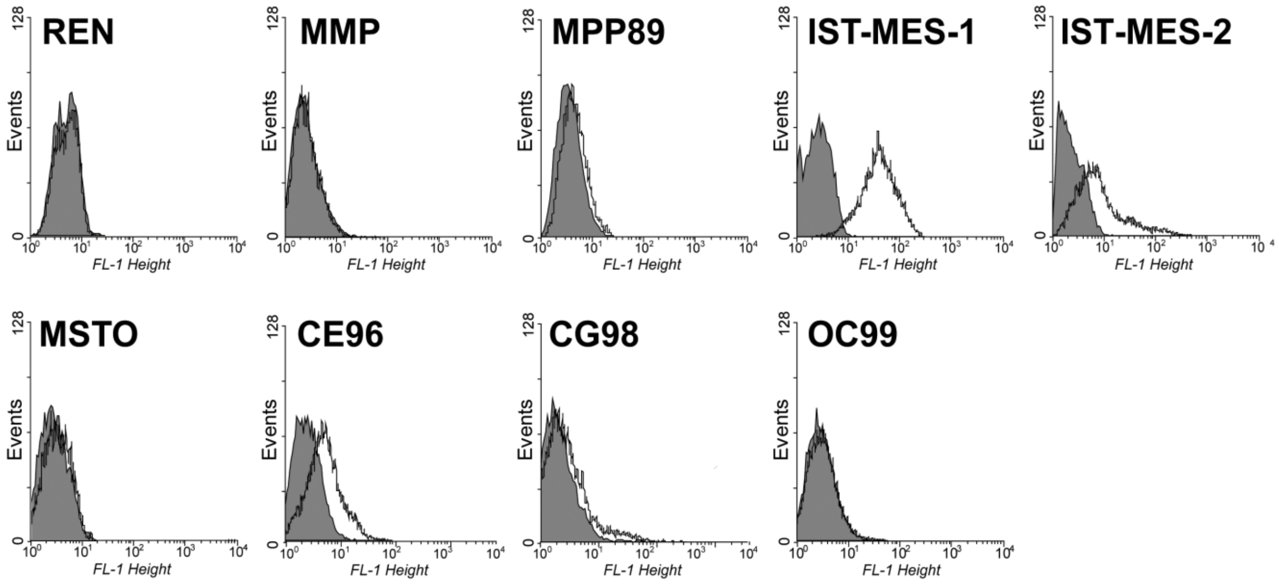

Figure S1: Flow cytometric analysis of the expression of CD38 in MPM cell lines. Cells were stained with the IB4 anti-CD38 antibody (white peaks) or isotype-matched murine monoclonal IgG (grey peaks) followed by F(ab')2-GaMIg-FITC. Fluorescence was analyzed using a FACSCalibur flow cytometer and CellQuest software. Ten thousand cells were considered for each analysis. *x*-axis = fluorescence intensity (FL-1 height), *y*-axis = number of cells (events).

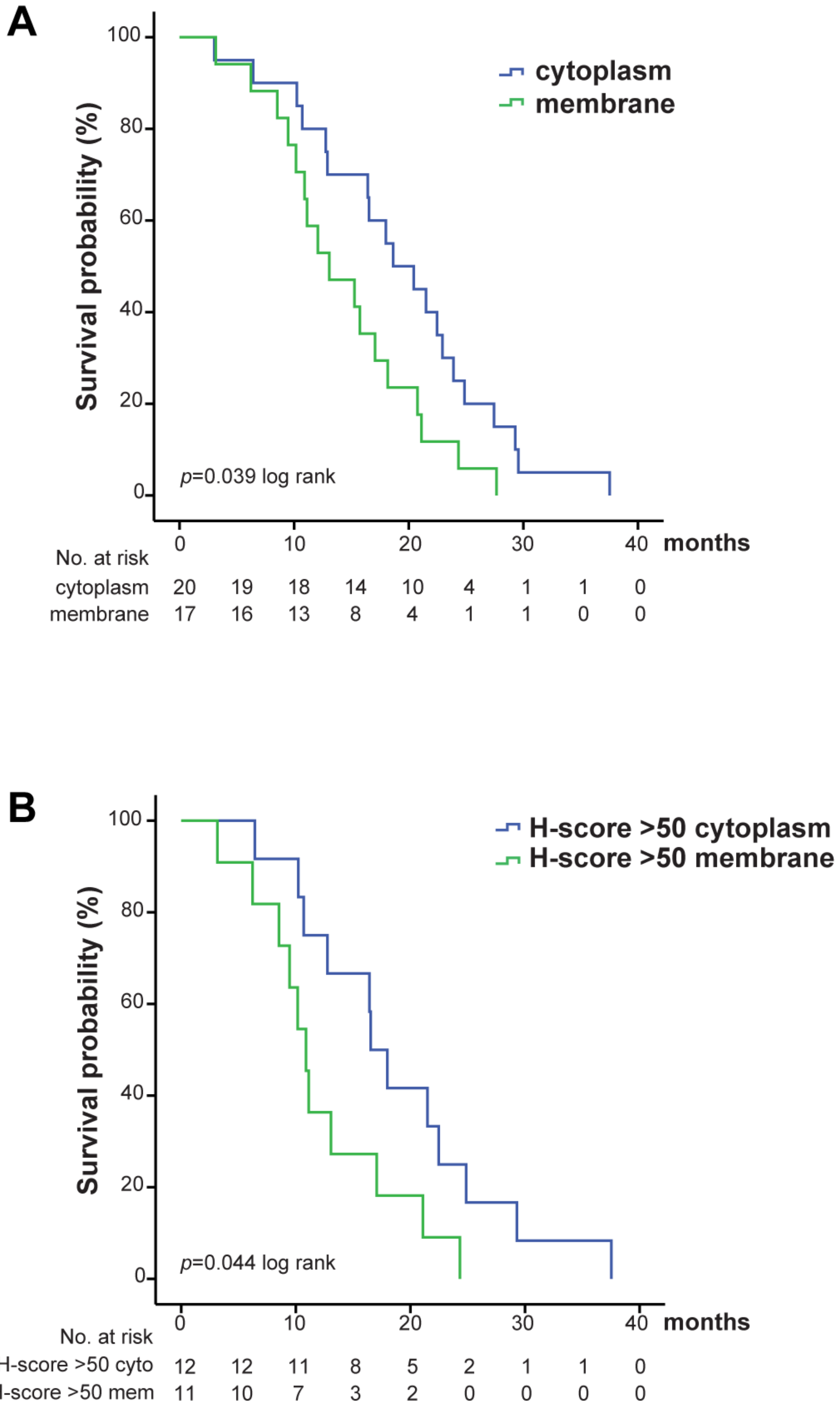

A
Survival probability (%)
100
80
60
40
20
0
cytoplasm
membrane
p=0.039 log rank
0
10
20
30
40
months
No. at risk
cytoplasm 20 19 18 14 10 4 1 1 0
membrane 17 16 13 8 4 1 1 0 0
B
Survival probability (%)
100
80
60
40
20
0
H-score >50 cytoplasm
H-score >50 membrane
p=0.044 log rank
0
10
20
30
40
months
No. at risk
H-score >50 cyto 12 12 11 8 5 2 1 1 0
H-score >50 mem 11 10 7 3 2 0 0 0 0

Figure S2: CD157 localization and clinical outcomes. (**A**) Kaplan-Meier analysis of survival in 44 patients with biphasic MPM derived from dichotomizing CD157 expression at membrane *versus* cytoplasmic localization. (**B**) Kaplan-Meier survival analysis of patients with biphasic MPM indicates that patients with CD157 H-score >50 and membrane localization had worse prognosis than patients with CD157 H-score >50 and cytoplasmic localization.

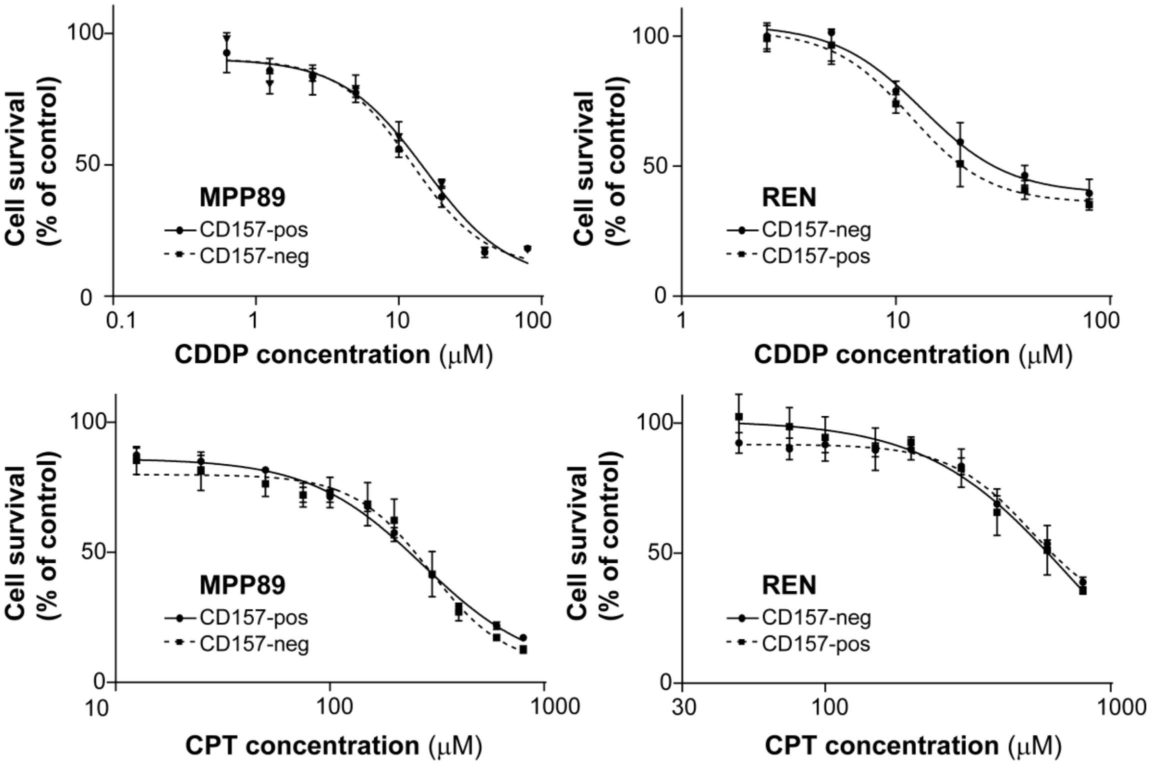

Figure S3: CD157 expression does not influence sensitivity to cisplatin and carboplatin in MPP89 and REN cells. Drug sensitivity assays were performed with increasing concentrations of CDDP (top panels) or CPT (bottom panels). Cells were treated for 48 h, then their viability was determined by MTT assays. Absorbance was read and each data was normalized to the respective scramble or mock vehicle control, and plotted. Results represent the mean ± SEM of at least three experiments performed in quadruplicate. A two-tailed unpaired t test was used to compare the viability of each CD157-positive *versus* CD157-negative cell line at each concentration of CDDP and CTP.

Supplementary Table S1: **Clinical features of 81 surgical MPM patients.**

**Clinical variables**	**No. of cases (%)**
**Sex**	
Male	60 (74.1)
Female	21 (25.9)
**Age at surgery, years**	
<55 (mean value)	33 (40.7)
≥55	48 (59.3)
**Histological type**	
Epithelioid	37 (45.7)
Biphasic	44 (54.3)
**Asbestos occupational exposure**	
No	40 (49.4)
Yes	41 (50.6)
**Disease stage, IMIG**	
I	2 (2.5)
II	9 (11.1)
III	41 (50.6)
IV	29 (35.8)
**Surgical treatment**	
P/D*	25 (30.9)
EPP°	56 (69.1)
**Resection margin status**	
radical (R0)	37 (45.7)
non radical (R1)	44 (54.3)
**Post-surgical chemotherapy**	
No	40 (49.4)
Yes	41 (50.6)
**CD157 expression**	
positive	69 (85.2)
negative	12 (14.8)
**CD157 H-score**	
≤50 (median value)	41 (50.6)
>50	40 (49.4)
**CD157 localization (in positive n=69)**	
cytoplasm	36 (52.2)
membrane	33 (47.8)

*P/D =pleurectomy/decortication (P/D)

°EPP= extrapleural-pneumonectomy (EPP)

Supplementary Table S2: CD157 expression and prevalent localization in patients with sarcomatoid MPM.

Patient ID	Age/Sex	CD157 H-score	CD157 localization
1-04AA	66/M	0	//
2-04CU	66/M	0	//
3-04DG	73/F	10	cytoplasm
4-05AF	71/M	0	//
5-05AA	73/M	0	//
6-05GF	70/M	10	cytoplasm
7-05BL	78/M	30	membrane
8-05ML	74/M	50	membrane
9-05CM	73/M	40	cytoplasm
10-06PE	73/M	20	cytoplasm
11-06BA	62/M	0	//
12-06GG	68/M	0	//
13-07CE	75/M	0	//
14-07DM	72/M	40	membrane
15-08BG	73/M	0	//
16-08SG	55/M	10	cytoplasm
17-08LA	76/F	20	membrane
18-09BC	71/M	0	//
19-10ZP	76/M	0	//
20-10BG	85/M	0	//

Supplementary Table S3: **Clinical variables of 81 MPM patients and their association with CD157 expression**

	All cases			
	**Total**	**CD157 H-score ≤50**	**CD157 H-score >50**	***p***
**Mesotheliomas**	81	41	40	
**Sex**				*0.08*
Male	60	34	26	
Female	21	7	14	
**Age at surgery (years)**				*1*
<55 (mean value)	33	17	16	
≥55	48	24	24	
**Histological type**				*0.657*
Epithelioid	37	20	17	
Biphasic	44	21	23	
**Asbestos occupational exposure**				*1*
No	40	20	20	
Yes	41	21	20	
**Disease stage**				*0.492*
I/II/III	52	38	24	
IV	29	13	16	
**Survival (months)**				*0.871*
**Median survival time**	18.00	18.633	16.533	

P values were determined using two-sided Fisher exact test.
